# Public Attitudes About the Use of Gene Therapy in Mainland China

**DOI:** 10.1001/jamanetworkopen.2023.28352

**Published:** 2023-08-11

**Authors:** Yiqi Li, Xinyue Zhang, Ze Xiang, Tianle Chen, Zihao Hu, Kexin Yang, Xinying Sun, Yibo Wu, Jian Wu

**Affiliations:** 1 Zhejiang University School of Medicine, Hangzhou, Zhejiang, China; 2 School of Health Policy and Management, Chinese Academy of Medical Sciences and Peking Union Medical College, Beijing, China; 3 School of Mathematical Sciences, Zhejiang University, Hangzhou, China; 4 School of Public Health, Peking University, Beijing, China; 5 Department of Laboratory Medicine, The Affiliated Suzhou Hospital of Nanjing Medical University, Suzhou Municipal Hospital, Gusu School, Nanjing Medical University, Suzhou, Jiangsu, China

## Abstract

**Importance:**

In addition to technical barriers, public attitudes about the use of gene therapy have an important association with the clinical implementation of gene therapy.

**Objective:**

To investigate the factors associated with public acceptance of gene therapy among individuals in China.

**Design, Setting, and Participants:**

This cross-sectional study used data from a survey conducted among 21 880 individuals in mainland China from June 20 to August 31, 2022.

**Main Outcomes and Measures:**

Stepwise linear regression was used to analyze factors associated with public acceptance of gene therapy in 5 key areas: basic personal information (gender, region, age, and educational level), family situation (marital status, children, and cousins), economic status (assets, debts, and insurance coverage), health knowledge (health literacy score and media use), and physical health status (chronic illness, cancer, European Quality of Life 5-Dimension 5-Level version [EQ-5D-5L] score, and Brief Illness Perception Questionnaire [BIPQ] score). Acceptance scores were calculated based on a visual analog scale (range, 0-100, with higher scores indicating higher acceptance of gene therapy). Further subgroup analysis was carried out in different age subgroups and populations with or without chronic diseases.

**Results:**

A total of 21 880 participants (mean [SD] age, 39.4 [18.9] years; 10 947 female participants [50.0%]; 10 933 male participants [50.0%]) were analyzed in this study. The mean (SD) acceptance score of gene therapy in the survey was 60.56 (27.60). Compared with people aged 60 years or older, those aged 12 to 18 years had higher acceptance of gene therapy (β = 1.48 [95% CI, 0.09-2.88]), while groups aged 19 to 30 years (β = −3.43 [95% CI, −4.80 to −2.07]), 31 to 44 years (β = −1.44 [95% CI, −2.76 to −0.12]), and 45 to 59 years (β = −2.05 [95% CI, −3.27 to −0.83]) had lower acceptance. Compared with people living in Eastern China, those in Central China had lower acceptance of gene therapy (β = −1.58 [95% CI, −2.54 to −0.62]), while those in Western China had higher acceptance (β = 0.92 [95% CI, 0.09-1.76]). Higher educational level (undergraduate or above vs junior high or below) was associated with higher acceptance of gene therapy (β = 1.56 [95% CI, 0.49-2.63]). Number of properties owned was also associated with higher acceptance of gene therapy (2 vs 0: β = 2.38 [95% CI, 1.04-3.72]; ≥3 vs 0: β = 4.66 [95% CI, 2.92-6.39]). Diagnosis of chronic disease was associated with lower acceptance of gene therapy (β = −17.86 [95% CI, −20.49 to −15.24]), while diagnosis of cancer was associated with higher acceptance (β = 6.99 [95% CI, 1.84-12.14]). Higher BIPQ score (β = 0.40 [95% CI, 0.34-0.45]), higher health literacy score (β = 0.70 [95% CI, 0.62-0.78]), and media use (β = 0.49 [95% CI, 0.41-0.57]) were all associated with high acceptance of gene therapy, while a higher EQ-5D-5L score was associated with lower acceptance (β = −0.29 [95% CI, −0.47 to −0.11]). For older people, being in debt, not having health insurance, and the EQ-5D-5L score were uniquely relevant factors. For people with chronic disease, having an undergraduate degree or higher, a diagnosis of cancer, and the BIPQ score were uniquely relevant factors.

**Conclusions and Relevance:**

These results suggest that basic personal information, economic status, health knowledge, and physical health status were the main factors associated with the acceptance of gene therapy. Improving the health literacy of the population and promoting trust in gene therapy may be effective ways to increase the acceptance of gene therapy. Poorer economic levels and worse disease states may reduce the public’s willingness to accept gene therapy.

## Key Points

**Question:**

What factors are associated with the level of public acceptance of gene therapy in mainland China?

**Findings:**

This cross-sectional study of 21 880 participants found that age, region, educational level, economic level, physical health, health literacy, and media use were associated with the level of gene therapy acceptance.

**Meaning:**

This study suggests that improving the health literacy of the population and promoting trust in gene therapy may be effective ways to increase the acceptance of gene therapy, while poorer economic levels and worse disease states may reduce the public’s willingness to accept gene therapy.

## Teaser

This cross-sectional study investigates the factors associated with public acceptance of gene therapy among individuals in China.

## Introduction

Gene therapy is a promising and potentially curative treatment for diseases that are refractory to conventional therapies.^1^ The core concept of gene therapy revolves around the transfer of genetic material into a patient’s cells, with the aim of correcting or countering a disease state.^2,3^ The primary objective of gene therapy is to address the underlying cause of a disease at the genetic level, rather than to simply manage its symptoms.^4^

The possibilities of treating previously untreatable diseases have engaged researchers to further discover gene therapy by means of rapidly evolving clinical practice and methods.^5,6,7,8,9,10,11,12,13,14^ However, as a complicated treatment, gene therapy is facing numerous uncertainties and challenges in its clinical implementation.^15,16^ Despite the potential of gene therapy to revolutionize medicine, there are still many intrinsic risks and challenges to the efficacy and accuracy of targeted delivery.^17^ There have been several reports of severe adverse events associated with gene therapy,^5,6,7,18,19,20^ which have raised concerns among both researchers and the public regarding ethical, social, and safety aspects of gene therapy. Such occurrences may compromise public confidence in gene therapy. China has been investing in significant efforts in the field of gene therapy and is now experiencing a period of rapid advancement^21^; the country has a relatively broad target audience for gene therapy. Public perceptions of gene therapy are significantly associated with the Chinese gene therapy market and its laws and regulations.

Understanding the public’s concerns and opinions about gene therapy is vital for managing the ethical and social challenges that could surface during its implementation. To explore the acceptance of gene therapy among the Chinese public, we conducted a cross-sectional survey in mainland China. We explore the factors that are significantly associated with the public’s acceptance of gene therapy in mainland China, which may provide valuable insights into possible obstacles to or facilitators of clinical applications of gene therapy.

## Methods

### Data Collection

This study is a population-based cross-sectional study conducted in Mainland China from June 20 to August 31, 2022. The study included 148 cities, 202 districts or counties, 390 townships or streets, and 780 communities or villages across 23 provinces, 5 autonomous regions, and 4 municipalities. Stratified random sampling was used at the provincial, community, or village level, while nonproportional quota sampling was used at the community, village, or individual level. Of 31 449 collected questionnaires, 30 505 met the qualification criteria after performing logical checks, and 21 916 remained after adjustment using quota sampling based on Chinese demographic characteristics.^22^ Of these 21 916 questionnaires, 36 were disregarded because the respondents had been outside mainland China for the past 3 months. In the final statistical analysis, a total of 21 880 data points were included (eFigure 1 in Supplement 1). The research protocol was approved by the Shaanxi Health Culture Research Center ethics review board and is currently registered in the Chinese Clinical Trial Registry (registration number ChiCTR2200061046). Written informed consent was obtained from all participants prior to their participation in the survey. This study followed the Strengthening the Reporting of Observational Studies in Epidemiology (STROBE) reporting guideline.

The survey was conducted anonymously. Investigators conducted the survey by distributing electronic questionnaires face-to-face with respondents. The questionnaire was designed on the Wenjuanxing platform.^23^ If face-to-face interviews were not suitable due to personal reasons (eg, respondents surveyed who are isolated at home due to SARS-CoV-2 or who are unable to complete the questionnaire by themselves due to physical reasons), then the investigators used online communication platforms to conduct video surveys.

The survey included participants who met the following inclusion criteria: (1) aged 12 years or older, (2) Chinese nationality, (3) permanent resident of China (out of country for no more than 1 month per year), (4) voluntarily agreed to participate in the study and signed an informed consent form, (5) able to complete the online questionnaire independently or with the assistance of the investigator, and (6) capable of comprehending the meaning of each item on the questionnaire. The exclusion criteria comprised the following: (1) having mental impairment or disability, (2) having cognitive impairment, (3) participation in other similar research projects, and (4) unwillingness to cooperate. The survey questionnaire used in this study underwent 38 expert consultations and 3 presurveys to guarantee the results’ validity.

### Research Instruments

We used visual analogue scale scores to assess public acceptance of gene therapy (range, 0-100). A higher score indicates higher acceptance of gene therapy. The factors associated with people’s acceptance of gene therapy are complex and multifaceted. Possible relative factors included (1) basic personal information (gender, region, age, and educational level), (2) family situation (marital status, children, and cousins), (3) economic status (assets, debts, and insurance coverage), (4) health knowledge (Short-Form Health Literacy Questionnaire, 12-item version [HLS-SF12] score,^24^ and media use), and (5) physical health status (chronic illness, cancer, European Quality of Life 5-Dimension 5-Level version [EQ-5D-5L] score,^25^ and Brief Illness Perception Questionnaire [BIPQ] score^26^) (eMethods in Supplement 1).

Participants’ quality of life and self-perceived health were assessed with the EQ-5D-5L across 5 dimensions: mobility, self-care, usual activities, pain and discomfort, and anxiety and depression.^25^ Scores ranged from 1 to 5 on a Likert-type scale, with a maximum total score of 25 and higher scores indicating poorer quality of life. In this study, the Cronbach α coefficient for the EQ-5D-5L scale was 0.811.

The HLS-SF12, used to measure health literacy, evaluated participants’ abilities to discover, understand, evaluate, and apply health literacy–related information.^24^ Response options for each item ranged from 1 to 4 on a Likert-type scale with 1 indicating very difficult and 4 indicating very easy, with a maximum total score of 48 and higher scores indicating higher levels of health literacy. In this study, the Cronbach α coefficient for the HLS-SF12 scale was 0.938.

The BIPQ evaluated patients’ perception of their illness based on 9 questions.^26^ The questionnaire was divided into 4 major categories: cognitive status (items 1-5), emotional status (items 6 and 7), comprehension (item 8), and causal perception (item 9). Items 1 to 8 were scored using the Likert method, with scores ranging from 0 to 10. The total score was calculated by summing the scores for all 9 items, with higher scores indicating that the patient perceived their illness to be more threatening. In this study, the Cronbach α coefficient for the BIPQ was 0.762.

Media use was a scale developed for this study to assess individuals’ media consumption behaviors, based on relevant literature.^27,28^ The scale included 6 items that correspond to 6 different types of media use behavior: social communication, self-presentation, social action (such as advocacy and promoting justice), leisure and entertainment, information acquisition through media, and commercial transactions. Each item was scored using a Likert-type 5-point scale, ranging from 1 (never used) to 5 (always used). The total score ranges from 6 to 30, with higher scores indicating more frequent media use. In this study, the Cronbach α coefficient for the media use scale was 0.872.

### Statistical Analysis

The collected data were analyzed by SPSS for Windows, version 26.0 (SPSS Inc). First, the 1-way analysis of variance was used to explore the association of individual factors with outcomes. Then, significantly different factors and theoretically correlated factors were subsequently incorporated into a subsequent forward stepwise linear regression. Further subgroup analysis was performed (subgroups by age and chronic diseases). Multicollinearity tests and score tests for the proportional odds assumption were performed to determine the analytical model. All *P* values were from 2-sided tests, and results were deemed statistically significant at *P* < .05.

## Results

### Sociological Characteristics of the Population

A total of 21 880 participants (mean [SD] age, 39.4 [18.9] years; 10 933 male participants [50.0%] and 10 947 female participants) were analyzed in this study (Table 1). A total of 3214 participants (14.7%) were aged 12 to 18 years, 5628 (25.7%) were aged 19 to 30 years, 4319 (19.7%) were aged 31 to 44 years, 4605 (21.0%) were aged 45 to 59 years, and 4114 (18.8%) were aged 60 years or older. Among the sample, 13 396 people (61.2%) had been married (including married, divorced, and widowed), and 8484 (38.8%) were not married. A total of 5652 people (25.8%) had a diagnosis of a chronic disease, of whom 108 (0.5% of the full sample and 1.9% of those with chronic disease) had a diagnosis of cancer. In terms of educational level, junior high or below, senior high or specialty education, and undergraduate or above each accounted for about one-third of the population.

**Table 1.** Descriptive Statistics on the Acceptance of Gene Therapy Among Study Participants

Characteristic	No. (%)	Acceptance score, mean (SD)	*F* value	*P* value
Total	21 880 (100)	60.56 (27.60)	NA	NA
Gender				
Female	10 947 (50.0)	60.80 (26.77)	1.71	.19
Male	10 933 (50.0)	60.31 (28.41)		
Region				
Eastern China	7203 (32.9)	61.14 (27.67)	14.14	<.001
Central China	5359 (24.5)	58.82 (28.89)		
Western China	9318 (42.6)	61.11 (26.75)		
Age group, y				
12-18	3214 (14.7)	64.12 (27.04)	21.34	<.001
19-30	5628 (25.7)	60.71 (27.69)		
31-44	4319 (19.7)	60.94 (28.36)		
45-59	4605 (21.0)	59.24 (28.36)		
≥60	4114 (18.8)	58.63 (25.93)		
Highest educational level				
Junior high or below	6960 (31.8)	58.80 (26.78)	33.89	<.001
Senior high or specialty education	7688 (35.1)	60.26 (28.04)		
Undergraduate or above	7232 (33.1)	62.57 (27.78)		
Marital status				
Unmarried	8484 (38.8)	62.10 (27.51)	43.27	<.001
Married	13 396 (61.2)	59.58 (27.62)		
Have children				
No	9455 (43.2)	62.08 (27.35)	50.87	<.001
Yes	12 425 (56.8)	59.40 (27.74)		
Have cousin(s)				
No	5861 (26.8)	62.03 (27.37)	22.84	<.001
Yes	16 019 (73.2)	60.02 (27.67)		
No. of properties owned				
0	2478 (11.3)	58.33 (29.62)	42.46	<.001
1	13 487 (61.6)	59.60 (27.00)		
2	4394 (20.1)	62.76 (27.37)		
≥3	1521 (7.0)	66.32 (29.03)		
Chronic disease diagnosis				
No	16 228 (74.2)	61.05 (27.57)	20.05	<.001
Yes	5652 (25.8)	59.14 (27.65)		
Cancer diagnosis				
No	21 772 (99.5)	60.52 (27.59)	6.98	.008
Yes	108 (0.5)	67.56 (29.54)		
Have debt				
No	13 742 (62.8)	60.46 (27.34)	0.50	.48
Yes	8138 (37.2)	60.73 (28.05)		
Have medical insurance				
No	1669 (7.6)	60.45 (29.08)	0.02	.88
Yes	20 211 (92.4)	60.57 (27.48)		
Investigation format				
Questionnaire	21 079 (96.3)	60.41 (27.60)	15.57	<.001
Online interviews	801 (3.7)	64.33 (27.55)		

Abbreviation: NA, not applicable.

### Acceptance of Gene Therapy and Associated Factors

The mean (SD) acceptance score for gene therapy in this study was 60.56 (27.60). More than half the study sample exhibited an acceptance level of 50 or above toward gene therapy (eFigure 2 in Supplement 1). The Figure displays the mean acceptance levels by province in mainland China. The results demonstrated that age, region, highest level of education, economic level (property ownership), physical health status (cancer diagnosis, BIPQ score, and EQ-5D-5L score), and health knowledge (HLS-SF12 score and media use) were all significant factors associated with gene therapy acceptance (Table 2).

**Figure.**
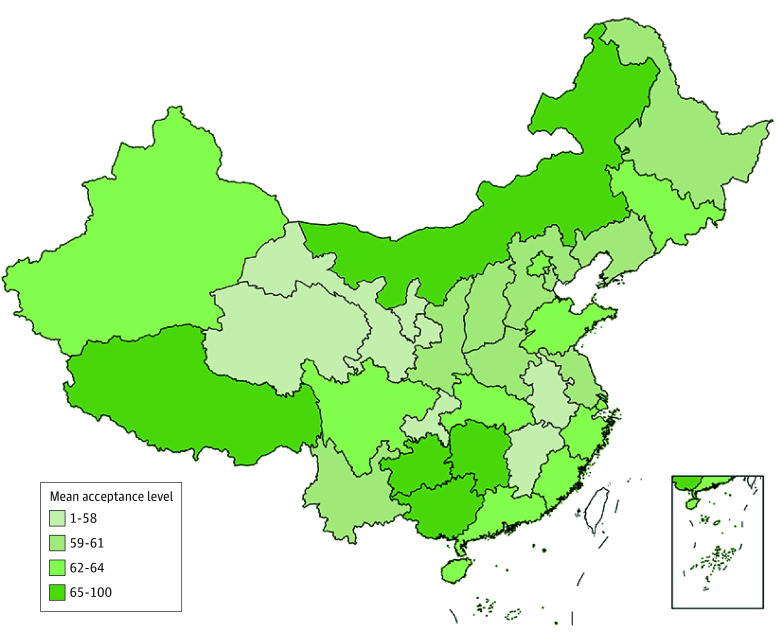
Mean Gene Therapy Acceptance Levels by Province in Mainland China Scores can range from 0 to 100. Higher scores indicate higher acceptance of gene therapy.

**Table 2.** Stepwise Regression Analysis of Factors Associated With the Acceptance of Gene Therapy

Variable	Unstandardized coefficient B		Standardized coefficient β	*t* Value	*P* value
	Mean (SE)	95% CI			
Constant	34.74 (1.21)	32.37 to 37.11	NA	28.705	<.001
Gender (reference: female)					
Male	−0.02 (0.36)	−0.73 to 0.70	0.00	−0.044	.97
Age group (reference: ≥60), y					
12-18	1.48 (0.71)	0.09 to 2.88	0.02	2.082	.04
19-30	−3.43 (0.70)	−4.80 to −2.07	−0.05	−4.923	<.001
31-44	−1.44 (0.67)	−2.76 to −0.12	−0.02	−2.139	.03
45-59	−2.05 (0.62)	−3.27 to −0.83	−0.03	−3.301	<.001
Region (reference: Eastern China)					
Central China	−1.58 (0.49)	−2.54 to −0.62	−0.02	−3.228	.001
Western China	0.92 (0.43)	0.09 to 1.76	0.02	2.161	.03
Highest educational level (reference: junior high or below)					
Senior high or specialty education	−0.45 (0.48)	−1.40 to 0.50	−0.01	−0.933	.35
Undergraduate or above	1.56 (0.55)	0.49 to 2.63	0.03	2.846	.004
No. of properties owned (reference: 0)					
1	0.24 (0.60)	−0.93 to 1.40	0.00	0.395	.69
2	2.38 (0.68)	1.04 to 3.72	0.03	3.483	<.001
≥3	4.66 (0.89)	2.92 to 6.39	0.04	5.260	<.001
Have chronic disease diagnosis (reference: no)					
Yes	−17.86 (1.34)	−20.49 to −15.24	−0.28	−13.345	<.001
Have cancer diagnosis (reference: no)					
Yes	6.99 (2.63)	1.84 to 12.14	0.02	2.660	.008
Investigation format (reference: questionnaire)					
Online interviews	4.03 (0.98)	2.12 to 5.95	0.03	4.133	<.001
BIPQ score	0.40 (0.03)	0.34 to 0.45	0.30	14.053	<.001
EQ-5D-5L score	−0.29 (0.09)	−0.47 to −0.11	−0.02	−3.174	.002
HLS-SF12 score	0.70 (0.04)	0.62 to 0.78	0.13	18.023	<.001
Media use score	0.49 (0.04)	0.41 to 0.57	0.10	12.102	<.001

Abbreviations: BIPQ, Brief Illness Perception Questionnaire; EQ-5D-5L, European Quality of Life 5-Dimension 5-Level version; HLS-SF12, Short-Form Health Literacy Questionnaire, 12-item version; NA, not applicable.

Compared with people aged 60 years or older, adolescents (aged 12-18 years; β = 1.48 [95% CI, 0.09-2.88]) had higher acceptance of gene therapy, while young people (aged 19-30 years; β = –3.43 [95% CI, −4.80 to −2.07]), middle-aged younger people (aged 31-44 years; β = −1.44 [95% CI, −2.76 to −0.12]), and middle-aged older people (aged 45-59 years; β = −2.05 [95% CI, −3.27 to −0.83]) had lower acceptance toward gene therapy (Table 2; eFigure 3 in Supplement 1). In contrast to populations residing in Eastern China, those living in Central China (β = −1.58 [95% CI, −2.54 to −0.62]) had lower acceptance of gene therapy, whereas the acceptance was higher among people living in Western China (β = 0.92 [95% CI, 0.09-1.76]). A higher educational level (undergraduate or above; β = 1.56 [95% CI, 0.49-2.63]) was positively correlated with a higher acceptance of gene therapy. In addition, compared with those who owned 0 properties, people who owned more property (which we infer to be indicative of greater wealth) (2 properties; β = 2.38 [95% CI, 1.04-3.72]; ≥3; β = 4.66 [95% CI, 2.92-6.39]) tended to be more receptive toward gene therapy. Those with a diagnosis of chronic disease (β = −17.86 [95% CI, −20.49 to −15.24]) showed significantly lower levels of acceptance of gene therapy, while those with a diagnosis of cancer (β = 6.99 [95% CI, 1.84-12.14]) showed significantly higher levels of acceptance toward gene therapy compared with the undiagnosed population.

The results also suggested that higher BIPQ score (β = 0.40 [95% CI, 0.34-0.45]), higher HLS-SF12 score (β = 0.70 [95% CI, 0.62-0.78]), and media use (β = 0.49 [95% CI, 0.41-0.57]) were positively associated with acceptance or gene therapy. We also observed that higher EQ-5D-5L score (β = −0.29 [95% CI, −0.47 to −0.11]) indicated poorer health states and lower acceptance of gene therapy.

### Factors Associated With Acceptance of Gene Therapy Among Middle-Aged and Elderly Populations

The results of the whole population regression showed that age was a significant factor of interest, so we further analyzed different age subgroups (Table 3). Region was an independent factor for all age groups, with people aged 45 years or older from Central China holding a lower level of acceptance of gene therapy than those aged 45 years or older from Eastern China, and people aged 31 to 44 years from Western China holding a higher level of acceptance than those aged 31 to 44 years from Eastern China. For those aged 31 to 59 years, educational level also correlated with the acceptance of gene therapy, with those with a bachelor’s degree or higher having a higher acceptance compared with those with a junior high or lower level of education. For those aged 60 years or older, there was no significant correlation between educational level and acceptance of gene therapy. In terms of property ownership, the acceptance of gene therapy was associated with higher economic levels (ie, ownership of more properties) among those aged 31 to 59 years, while acceptance of gene therapy was not associated with higher economic levels for those aged 60 years or older. However, being in debt and having health insurance were associated with acceptance of gene therapy for people aged 60 years or older, and unlike other age groups, those aged 60 years or older with a relatively poorer economic level (being in debt and not having health insurance) had a higher acceptance of gene therapy. Among individuals aged 31 to 44 years and those aged 60 years or older, online interview respondents had a higher willingness to accept gene therapy than offline questionnaire respondents. Diagnosis of chronic disease was a significant independent factor across all age groups, with those with chronic disease having lower acceptance of gene therapy. In addition, BIPQ, HLS-SF12, and media use scores were positively associated with acceptance of gene therapy at all ages, while the EQ-5D-5L score was associated with lower acceptance of gene therapy only among those aged 60 years or older.

**Table 3.** Analysis of Differences in Linear Regression Models by Age Subgroup

Variable	31-44 y			45-59 y				≥60 y			
	Standardized coefficient β	95% CI^a^	*P* value	Standardized coefficient β	95% CI^a^	*P* value	Standardized coefficient β		95% CI^a^	*P* value
Constant	NA	29.16 to 42.23	<.001	NA	14.35 to 27.83	<.001	NA		43.88 to 55.19	<.001
Gender (reference: female)										
Male	−0.02	−2.62 to 0.72	.27	−0.02	−2.71 to 0.49	.17	0.00		−1.28 to 1.74	.77
Region (reference: Eastern China)										
Central China	0.02	−0.99 to 3.33	.29	−0.04	−4.74 to −0.43	.02	−0.08		−7.97 to −3.37	<.001
Western China	0.05	0.85 to 4.92	.005	−0.01	−2.67 to 1.21	.46	0.00		−1.91 to 1.49	.81
Highest educational level (reference: junior high or below)										
Senior high or specialty education	−0.01	−2.58 to 1.93	.78	0.01	−1.05 to 2.72	.39	−0.02		−3.30 to 0.46	.14
Undergraduate or above	0.04	0.18 to 4.75	.03	0.04	0.34 to 4.90	.02	0.01		−2.41 to 3.42	.73
No. of properties owned (reference: 0)										
1	−0.01	−3.53 to 2.01	.59	0.02	−1.61 to 4.58	.35	−0.03		−4.70 to 0.73	.15
2	0.04	−0.01 to 6.45	.05	0.03	−1.41 to 5.44	.25	−0.01		−3.74 to 2.69	.75
≥3	0.04	0.03 to 8.67	.05	0.04	0.67 to 9.83	.02	0.03		−0.89 to 8.57	.11
Have chronic disease diagnosis (reference: no)										
Yes	−0.19	−20.40 to −8.06	<.001	−0.28	−21.64 to −11.88	<.001	−0.64		−38.19 to −28.43	<.001
Have debt (reference: no)										
Yes	0.01	−0.95 to 2.47	.39	0.00	−1.85 to 1.56	.87	0.05		1.38 to 5.49	<.001
Have medical insurance (reference: no)										
Yes	−0.01	−5.15 to 2.72	.54	0.02	−0.71 to 7.45	.11	−0.05		−9.25 to −2.72	<.001
Investigation format (reference: questionnaire)										
Online interviews	0.03	0.46 to 11.18	.03	0.01	−3.14 to 7.50	.42	0.05		2.42 to 8.71	<.001
BIPQ score	0.23	0.24 to 0.51	<.001	0.32	0.30 to 0.51	<.001	0.61		0.52 to 0.71	<.001
EQ-5D-5L score	−0.03	−0.91 to 0.01	.06	0.01	−0.30 to 0.60	.52	−0.07		−1.05 to −0.40	<.001
HLS-SF12 score	0.11	0.44 to 0.79	<.001	0.18	0.83 to 1.19	<.001	0.13		0.45 to 0.78	<.001
Media use score	0.07	0.26 to 0.65	<.001	0.07	0.26 to 0.62	<.001	0.09		0.24 to 0.55	<.001

Abbreviations: BIPQ, Brief Illness Perception Questionnaire; EQ-5D-5L, European Quality of Life 5-Dimension 5-Level version; HLS-SF12, Short-Form Health Literacy Questionnaire, 12-item version; NA, not applicable.

^a^ The 95% CIs are of the unstandardized coefficient B.

### Factors Associated With Acceptance of Gene Therapy Among Populations With Chronic Diseases

The population with chronic diseases, including cancer and some hereditary illnesses, was a primary focus of gene therapy. To identify the key factors associated with acceptance of gene therapy among these patients, we conducted a detailed subgroup analysis.

Factors associated with acceptance of gene therapy differed significantly between the group with and the group without chronic diseases (Table 4). For the group without chronic diseases, there was lower acceptance of gene therapy in all other age groups compared with those aged 60 years or older, while in the group with chronic diseases, there was no significant difference among the group aged 19 to 30 years, the group aged 45 to 59 years and the the group aged 60 years or older. For the population with chronic diseases, higher level of education was associated with higher acceptance of gene therapy. Better economic status was associated with higher acceptance of gene therapy in both groups, with higher acceptance among those with 3 or more properties compared with those with no property in the groups with chronic diseases, and among those with 2 or more properties compared with those with no property in the group without chronic diseases. For the group without chronic diseases, online interview respondents had a higher acceptance of gene therapy, which did not reflect differences in the group with chronic diseases. The presence or absence of a cancer diagnosis and the BIPQ score were significantly correlated with acceptance of gene therapy among patients with chronic diseases. In addition, for both groups, HLS-SF12 and media use scores were positively associated with acceptance of gene therapy, and EQ-5D-5L scores were negatively associated with acceptance.

**Table 4.** Analysis of Differences in Linear Regression Models Between Subgroups With or Without Chronic Illness

Variable	With chronic illness				Without chronic illness			
	Standardized coefficient β	95% CI^a^	*P* value	Standardized coefficient β		95% CI^a^	*P* value
Constant	NA	13.06 to 23.08	<.001	NA		33.26 to 38.93	<.001
Gender (reference: female)							
Male	−0.01	−1.92 to 0.86	.45	0.00		−0.77 to 0.89	.88
Age group (reference: ≥60), y							
12-18	0.05	2.49 to 9.78	<.001	−0.02		−3.35 to −0.02	.05
19-30	0.01	−1.78 to 3.64	.50	−0.11		−8.12 to −4.77	<.001
31-44	0.04	0.61 to 5.43	.01	−0.07		−6.42 to −3.11	<.001
45-59	0.02	−0.50 to 3.23	.15	−0.08		−7.20 to −3.92	<.001
Region (reference: Eastern China)							
Central China	−0.04	−4.35 to −0.57	.01	−0.02		−2.25 to −0.03	.04
Western China	−0.03	−3.14 to 0.05	.06	0.03		0.82 to 2.78	<.001
Highest educational level (reference: junior high or below)							
Senior high or specialty education	0.02	−0.73 to 2.88	.24	−0.02		−2.13 to 0.11	.08
Undergraduate or above	0.06	1.95 to 6.21	<.001	0.01		−0.48 to 2.01	.23
No. of properties owned (reference: 0)							
1	−0.01	−2.95 to 1.99	.70	0.01		−0.79 to 1.86	.43
2	0.03	−0.61 to 4.94	.13	0.04		0.93 to 3.99	.002
≥3	0.04	1.17 to 8.21	.008	0.04		2.54 to 6.54	<.001
Have cancer diagnosis (reference: no)							
Yes	0.03	1.55 to 11.79	.01	NA			
Investigation format (reference: questionnaire)							
Online interviews	0.02	−1.04 to 5.58	.18	0.03		2.03 to 6.71	<.001
BIPQ score	0.21	0.39 to 0.50	<.001	NA			
EQ-5D-5L score	−0.03	−0.61 to −0.06	.02	−0.02		−0.49 to −0.01	.04
HLS-SF12 score	0.11	0.41 to 0.71	<.001	0.14		0.66 to 0.83	<.001
Media use score	0.08	0.25 to 0.56	<.001	0.09		0.40 to 0.59	<.001

Abbreviations: BIPQ, Brief Illness Perception Questionnaire; EQ-5D-5L, European Quality of Life 5-Dimension 5-Level version; HLS-SF12, Short-Form Health Literacy Questionnaire, 12-item version; NA, not applicable.

^a^ The 95% CIs are of the unstandardized coefficient B.

## Discussion

In this cross-sectional study, gender as a demographic factor was not associated with the acceptance of gene therapy, which is contrary to results of a previous study.^29^ This finding may be associated with the larger scope of our investigation and the larger sample size. In line with previous findings,^30,31^ acceptance of gene therapy was higher among people with a higher level of education. This finding may be associated with greater exposure to information about gene therapy and a better understanding of how gene therapy works. The HLS-SF12 results suggested that higher health literacy was positively correlated with acceptance of gene therapy. The understanding of gene therapy, or health knowledge, could be associated with a greater trust in medicine, which in turn could increase acceptance of new treatments.

Age was found to be an important factor associated with the public’s acceptance of gene therapy, which is different than the association between age and attitude toward gene therapy found in previous studies.^30,32,33^ Based on the association between age and chronic disease, we further analyzed subpopulations with and without chronic diseases and found that acceptance of gene therapy was different among different age groups. The reasons for this need to be further investigated.

Media use demonstrates public access to information. Higher media use may familiarize the public with gene therapy or other health issues, which could promote public trust in medicine and novel therapies. In addition, economic status (measured by the number of properties owned) was positively correlated with the acceptance of gene therapy. Because the cost of gene therapy in the current clinical application is relatively high,^34^ individuals who undergo gene therapy may be more likely to have more financial resources.

Diagnoses of chronic disease and cancer were also important relevant factors. Patients with chronic diseases may have more concerns about gene therapy, such as the complexity of the treatment and the potential dangers. However, due to the significant progress made in gene therapy in the field of cancer, patients with cancer may be more aware of the benefits associated with gene therapy and therefore have a higher level of acceptance. Disease severity is directly associated with the patient’s attitude toward gene therapy.^35^ Patients with chronic disease with a higher BIPQ score could be more hopeful to recover and more willing to try new therapies to treat difficulties that are not adequately addressed by current conventional treatments. They may also be more confident that they can withstand the risks and uncertainties associated with treatment. However, we found that people with higher EQ-5D-5L scores were less receptive to gene therapy, which needs to be further studied in the future.

Age subgroup analysis results suggest that for middle-aged people (aged 31-59 years), reducing the cost of other products in clinical trials and enhancing health knowledge and understanding of gene therapy can help improve their acceptance of gene therapy. For the elderly population (aged ≥60 years) whose economic status was lower, we found higher levels of acceptance of gene therapy. This finding suggests that, based on economic level, older individuals have different viewpoints than young people and middle-aged people. Older people with higher a economic status may receive more conservative treatment rather than advanced therapy. For this reason, the demand for gene therapy among older patients without insurance and with chronic diseases deserves more attention in clinical trials.

Acceptance of gene therapy was lower among patients with chronic diseases than those without chronic diseases, while the acceptance level was significantly higher among patients with cancer. The higher acceptance level among the population with cancer compared with individuals with other chronic diseases may be due to the progress of gene therapy in the field of cancer (such as CAR [chimeric antigen receptor] T-cell therapy) on the one hand, and the greater urgency of cancer compared with other types of diseases on the other hand. As treatment options become more limited, people’s tolerance for risk increases.^36^ For people with chronic diseases, educational level was significantly correlated with acceptance of gene therapy, which was not demonstrated in the group without chronic diseases. Based on the results, professional medical staff can popularize the basic principles, treatment methods, advantages, disadvantages, and scope of gene therapy^37^ to improve the understanding of clinical trials of gene therapy and the trust of medical staff by patients with chronic diseases.

Gene therapy is facing not only the challenges of technical and scientific bottlenecks but also a lack of public knowledge. However, gene therapy may be the best choice for people with more limited treatment options. While breaking through the bottleneck of science and technology, we should also pay attention to the public’s knowledge about gene therapy, improving trust in gene therapy as a new treatment method. Gene therapy is not yet a good option for people with poorer economic status, less-severe disease, or more treatment options, and other therapies should be considered.

### Limitations

This study has several limitations that must be acknowledged. First, the research was conducted solely at the community level and did not include data analysis among hospitalized patients, which may limit the generalizability of the findings. Second, the questionnaire used in this study included self-report and self-assessment sections, which could introduce bias to the results. Third, our study used a cross-sectional design, which can only describe correlations rather than establish causality between factors. Future research is needed to focus on exploring causal relationships among the factors to enhance our understanding of gene therapy acceptance.

## Conclusion

This cross-sectional study provided insights into the acceptance of gene therapy across various age groups and regions in mainland China. The study findings indicated that basic personal information, economic status, health knowledge, and physical health status were the key factors associated with the acceptance of gene therapy. This research shows the level of acceptance of gene therapy within Chinese society, particularly among the elderly population and individuals with chronic illnesses. The results suggest that increasing access to accurate health knowledge through media channels and improving residents’ health literacy and awareness of gene therapy may positively affect their trust in this innovative therapeutic approach. Poorer economic levels and worse disease states may reduce the public’s willingness to accept gene therapy.

## Notes

Supplement 1.
eMethods.

eReferences.
**eFigure 1.** Flow Chart for Inclusion of Study Participants **eFigure 2.** Histogram of the Acceptance of Gene Therapy Distribution **eFigure 3.** Distribution of the Acceptance of Gene Therapy by Age

Supplement 2.
Data Sharing Statement
