# Supplementary material for: Public Attitudes About the Use of Gene Therapy in Mainland China
Source: JAMA Netw Open. 2023 Aug 11;6(8):e2328352. doi: 10.1001/jamanetworkopen.2023.28352 (PMC10422191; doi:10.1001/jamanetworkopen.2023.28352)
Supplement: Supplement 1. — eMethods. eReferences. eFigure 1. Flow Chart for Inclusion of Study Participants eFigure 2. Histogram of the Acceptance of Gene Therapy Distribution eFigure 3. Distribution of the Acceptance of Gene Therapy by Age [file jamanetwopen-e2328352-s001.pdf]

Supplementary Online Content

Li Y, Zhang X, Xiang Z, et al. Public attitudes about the use of gene therapy in mainland China. *JAMA Netw Open.* 2023;6(8):e2328352.
doi:10.1001/jamanetworkopen.2023.28352

eMethods.
eReferences.
eFigure 1. Flow Chart for Inclusion of Study Participants
eFigure 2. Histogram of the Acceptance of Gene Therapy Distribution
eFigure 3. Distribution of the Acceptance of Gene Therapy by Age

This supplementary material has been provided by the authors to give readers additional information about their work.

© 2023 Li Y et al. *JAMA Network Open.*

eMethods.

Research Instruments

In this survey, we used VAS (visual analogue scale) scores from 0 to 100 to assess public acceptance of gene therapy. Higher score indicates higher acceptance of gene therapy.

The EuroQol Five Dimensions Questionnaire (EQ-5D) was utilized in this study to gauge the respondents' quality of life and self-perceived health[1]. The five-level version EQ-5D-5L was used to evaluate the participants across five dimensions: mobility, self-care, usual activities, pain/discomfort, and anxiety/depression. The scores ranged from 1 to 5 (on a Likert-type scale), with a maximum total score of 25 and higher scores indicating poorer quality of life. In this study, the Cronbach's alpha coefficient for the EQ-5D scale was 0.811.

The Short-Form Health Literacy Questionnaire (HLS-SF) was employed to measure health literacy, which includes a 12-item version (HLS-SF12) in this study[2]. The HLS-SF12 evaluated participants' abilities to discover, understand, evaluate, and apply health literacy-related information. Response options for each item ranged from "very difficult" to "very easy" and assigned values from 1 to 4 on a Likert-type scale, with a maximum total score of 48. Higher scores indicated higher levels of health literacy. In this study, the Cronbach's alpha coefficient for the HLS-SF12 scale was 0.938.

The Brief Illness Perception Questionnaire (BIPQ) evaluated a patient's perception of their illness based on nine questions[3]. The questionnaire was divided into four major categories, namely cognitive status (items 1-5), emotional status (items 6 and 7), comprehension (item 8), and causal perception (item 9). Items 1-8 were scored using the Likert method, with scores ranging from 0 to 10. The total score was calculated by summing the scores for all nine items. A higher score indicated that the patient perceives their illness to be more threatening. In this study, the Cronbach's alpha coefficient for the BIPQ was 0.762.

© 2023 Li Y et al. *JAMA Network Open.*

Media Use was a scale developed for this study to assess individuals' media consumption behaviors, based on relevant literature[4,5]. The scale included six items that correspond to six different types of media usage behavior: social communication, self-presentation, social action (such as advocacy and promoting justice), leisure and entertainment, information acquisition through media, and commercial transactions. Each item was scored using a Likert-type five-point scale, ranging from 1 (never used) to 5 (always used). The total score ranges from 6 to 30, with higher scores indicating more frequent media use. In this study, the Cronbach's alpha coefficient for the Media Use scale was 0.872.

The division of mainland China in this study was based on the "Chinese Statistical Yearbook 2021"[6]. The eastern region included Beijing, Tianjin, Hebei, Liaoning, Shanghai, Jiangsu, Zhejiang, Fujian, Shandong, Guangdong, and Hainan; the central region included Shanxi, Jilin, Heilongjiang, Anhui, Jiangxi, Henan, Hubei, and Hunan; the western region included Nei Mongol, Chongqing, Guangxi, Sichuan, Guizhou, Yunnan, Tibet, Shaanxi, Gansu, Qinghai, Ningxia, Xinjiang.

Quota-sampled

The sample had been quota-sampled based on China's demographic characteristics, and the resulting distribution is as follows:(1) Age distribution: 12-18 year-olds accounted for approximately 15% of the sample; 19-25 year-olds accounted for approximately 16%; 26-30 year-olds accounted for approximately 10%; 31-40 year-olds accounted for approximately 1%; 41-50 year-olds accounted for approximately 17%; 51-60 year-olds accounted for approximately 12%; 61-70 year-olds accounted for approximately 11%; those over 71 years old accounted for approximately 7%. (2) Gender distribution: The male-to-female ratio in the sample was 1:1. (3) Urban-rural distribution: The urban-to-rural sample ratio was approximately 7:3.

© 2023 Li Y et al. *JAMA Network Open.*

eReferences.

1. Herdman M, Gudex C, Lloyd A, et al. Development and preliminary testing of the new five-level version of EQ-5D (EQ-5D-5L). *Qual Life Res*. 2011;20(10):1727-1736. doi:10.1007/s11136-011-9903-x

2. Duong TV, Aringazina A, Kayupova G, et al. Development and Validation of a New Short-Form Health Literacy Instrument (HLS-SF12) for the General Public in Six Asian Countries. *HLRP: Health Literacy Research and Practice*. 2019;3(2). doi:10.3928/24748307-20190225-01

3. Broadbent E, Petrie KJ, Main J, Weinman J. The Brief Illness Perception Questionnaire. *Journal of Psychosomatic Research*. 2006;60(6):631-637. doi:10.1016/j.jpsychores.2005.10.020

4. den Hamer AH, Konijn EA, Plaisier XS, Keijer MG, Krabbendam LC, Bushman BJ. The Content-based Media Exposure Scale (C-ME): Development and Validation. *Computers in Human Behavior*. 2017;72:549-557. doi:10.1016/j.chb.2017.02.050

5. Whyte W, Hennessy C. Social Media use within medical education: A systematic review to develop a pilot questionnaire on how social media can be best used at BSMS. *MedEdPublish*. 2017;6:83. doi:10.15694/mep.2017.000083

6. National Bureau of Statistics of China. Chinese Statistical Yearbook 2021. http://www.stats.gov.cn/sj/ndsj/2022/indexch.htm

© 2023 Li Y et al. *JAMA Network Open.*

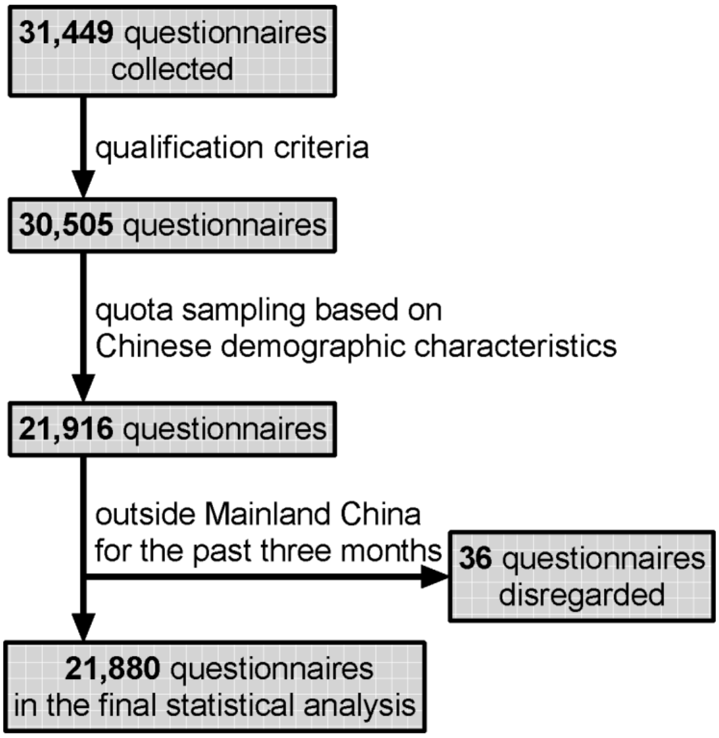

eFigure 1. Flow Chart for Inclusion of Study Participants

© 2023 Li Y et al. *JAMA Network Open.*

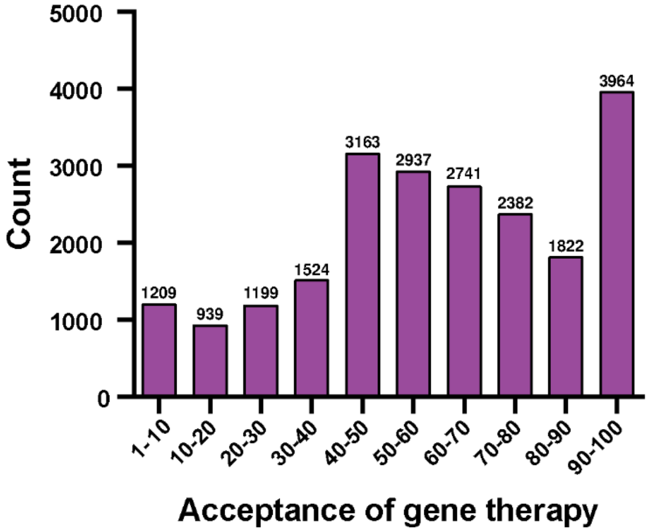

eFigure 2. Histogram of the Acceptance of Gene Therapy Distribution

© 2023 Li Y et al. *JAMA Network Open.*

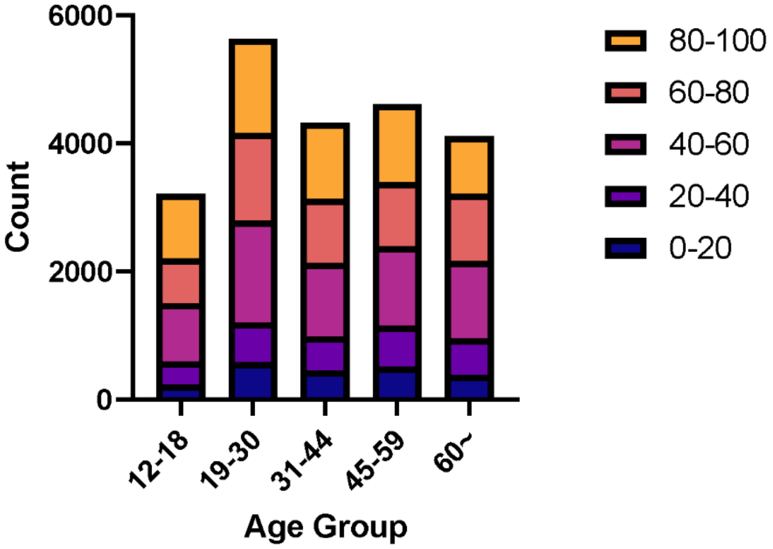

eFigure 3. Distribution of the Acceptance of Gene Therapy by Age

© 2023 Li Y et al. *JAMA Network Open.*
