# Supplementary material for: Public Attitudes About the Use of Gene Therapy in Mainland China
Source: JAMA Netw Open. 2023 Aug 11;6(8):e2328352. doi: 10.1001/jamanetworkopen.2023.28352 (PMC10422191; doi:10.1001/jamanetworkopen.2023.28352)
Supplement: Supplement 2. — Data Sharing Statement [file jamanetwopen-e2328352-s002.pdf]

Data Sharing Statement

Li. Public Attitudes About the Use of Gene Therapy in Mainland China. *JAMA Netw Open*. Published August 11, 2023. doi:10.1001/jamanetworkopen.2023.28352

Data
Data available: No
